# Editorial: Overcoming (in)visible barriers: gender, work and discrimination

**DOI:** 10.3389/fsoc.2026.1858426

**Published:** 2026-05-08

**Authors:** Yvonne Ehrstein, Stefano Maiani, Cláudia Andrade

**Affiliations:** 1 Department of Geography and Earth Sciences, Aberystwyth University, Aberystwyth, United Kingdom; 2 Edinburgh Business School, Heriot-Watt University, Edinburgh, United Kingdom; 3 Coimbra Education School, Polytechnic of Coimbra, Coimbra, Portugal; 4 inED Center for Research and Innovation in Education, Coimbra, Portugal

**Keywords:** gender discrimination, institutional inequality, intersectionality, subtle discrimination, workplace inequality

Legislative reforms, diversity initiatives, and equality frameworks have reduced many overt forms of gender inequality, but discrimination has not disappeared—it has evolved (Jones et al., 2017). Indeed, gender discrimination in today's workplace remains a central concern (Benschop, 2021; Kelan, 2020), increasingly sustained through subtle, and often invisible yet systemic mechanisms embedded within everyday workplace interactions and gendered organizational structures (Acker, 1990; Jones et al., 2017). In recent years, campaigns such as #MeToo have amplified both the visibility of gender discrimination in organizations and the role of social media in enabling collective action against workplace discrimination and inequality; a development that may also have contributed to the intensifying shift from blatant to more subtle forms of discrimination (Dipboye and Halverson, 2004; Quan-Haase et al., 2021).

This Research Topic documents different forms of gender discrimination in organizations across a range of countries and sociocultural contexts, shedding light on how dimensions of culture and context/regulation, and organization- and industry-level effects intertwine and shape discrimination outcomes. It assembles contributions that explore various sectoral settings, but importantly also presents articles that analyse aspects of workplace inequality through Global South and post-Soviet perspectives which are still too often marginalized in the sociology of work and organization studies.

It offers answers to primarily three interrelated questions. First, how does organizational discrimination persist in contemporary workplaces through both subtle and overt forms, as discrimination becomes less visible yet remains consequential for employees' career trajectories, wellbeing, and access to opportunities? Second, how do forms of subtle discrimination manifest within organizational settings, and through which mechanisms are they reproduced across different labor regimes? Third, what role do context-specific organizational structures and policy frameworks play in shaping discriminatory practices?

Across several contributions, discrimination emerges through institutionalized invisibility, with significant implications for employees' professional advancement (Isirabahenda et al., Menard; Spasova) and health outcomes (Bjerkestrand et al.). Interesting to note is that such forms of discrimination are produced not only through exclusion (e.g., lack in hiring or promotion denial), but also through the organization of time at work (Menard; Sardadvar). In this sense, contract durations and working hours shape directly who progresses and under what conditions—delaying advancement, extending precarity, and imposing uneven temporal norms—especially for women. Menard focuses on UK long-term researchers as a group of overlooked workers who remain in casualised roles for extended periods, often for over 8 years. Their career progression is systematically delayed despite sustained contributions, illustrating incisively how certain, common forms of academic employment normalize prolonged precarity as an academic “rite of passage” and defer (or hinder) recognition due to marginalization and invisibility. Analyzing marginalization and issues around in/visibility and time in a different sector, Sardadvar's study of cleaning work in Austria shows that even when cleaners' work—which is typically temporally invisible and performed at the margins of the day—is re-organized into daytime hours, ostensibly increasing visibility, new forms of subtle interaction-based discrimination such as “cold greetings” emerge. Visibility in the cleaning sector is then highly ambivalent; it has the potential to enhance recognition, but also enables additional surveillance and interpersonal monitoring. Taken together, these findings suggest that discrimination is not simply a matter of “being unseen”; it is also reproduced through the very forms of visibility made available to workers.

These dynamics are reinforced in highly standardized and performance-driven environments like the Romanian outsourced service sector, where often-invisible organizational routines, metrics, and emotional labor requirements embed gendered expectations into everyday work practices that often result in deskilling, burnout, and constrained career mobility (Isirabahenda et al.). As Yang et al. show on the other hand, when power imbalances are justified by traditional cultural norms around hierarchical gender roles such as those emphasized by Chinese Confucian culture, overt discrimination is more likely, as organizational cultures shaped by hierarchical authority and associated gendered norms can foster workplace bullying, disproportionately affecting women.

Two articles explore longstanding discriminatory perceptions and practices surrounding parenthood at work in post-socialist countries. Dancíková and Muter's comparative study of Poland and Slovakia demonstrates that it is not just mothers who are impacted by care expectations in gendered organizations. They show that fathers face subtle forms of discrimination when deviating from normative breadwinner roles, especially when they seek to take parental leave, though the extent varies depending on policy design. Their analysis demonstrates that policy details matter, but also that even well-intentioned policies can reproduce inequality if they fail to challenge underlying ideal worker/male breadwinner norms. In turn, Spasova's findings highlight the continued penalization of motherhood in the Bulgarian work context, particularly for younger women in precarious employment.

As Spasova shows for Bulgaria, and Baker et al. and Mathetha and Dhanpat demonstrate for the South-African mining industry and broader corporate leadership landscape, respectively, there is a tension between formal workplace diversity and equality commitments and the continuity of gendered forms of inequality and discrimination at work. A situation whereby progressive policy frameworks that have taken on board (a selection of) feminist ideas coexist with entrenched patriarchal norms and institutional barriers is not unique to labor markets and organizations operating in the Global South and Eastern Europe. Indeed, these dynamics align with the findings of US-focussed and global studies (Ehrstein et al., forthcoming), and notably also extend to younger cohorts entering the labor market, as Andrade et al. show for the Portuguese context, where gender continues to shape what is considered “decent work,” including working conditions, career advancement, and perceptions of fairness. Gabarrot's theoretical work confirms that these issues are sustained through seemingly neutral standards of competence and the “ideal worker,” which reflect dominant group norms and embed exclusion within everyday evaluative practices, often under the appearance of meritocracy.

The articles constituting this Research Topic also call for reconsidering intersectionality and its role in the complexification and reproduction of workplace discrimination and inequality, evidencing the usefulness of an intersectional frame of analysis across labor regimes (Cho et al., 2013). For minoritised British women workers of African, Asian, and Caribbean backgrounds, identity imposition processes—whereby intersecting racialised and gendered expectations constrain self-presentation at work—are being responded to via adaptation and ambivalence, but also resistance (Opara et al.). Beyond identity formation, an intersectional lens also shows discrimination becomes embodied as a health outcome: among Norwegian sexual and gender minority employees, intersecting statuses shape exposure to interpersonal discrimination, which—though partially mitigated by allyship—are linked to poorer self-rated health (Bjerkestrand et al.). Yet again in UK academia, age, gender, and, as part of the latter, caring responsibilities, position long-term researchers as simultaneously over-experienced and under-recognized—“too old” for precarity yet confined within it (Menard). In the cleaning sector in Austria and beyond, gender intersects with migrant status and class to normalize both invisibility and servitude within fissured workplaces (Sardadvar). Motherhood also emerges as a critical site where gendered disadvantage unfolds in layered, intersectional ways, shaped by age, ethnicity and class, and builds up over time for the women in Spasova's study.

Addressing discrimination dynamics requires moving beyond formal equality measures toward interventions that transform organizational cultures and evaluation practices, enabling more substantive and sustainable forms of workplace equity (Spasova; Baker et al.). This also involves cultivating everyday practices of support and accountability structures that has the potential to interrupt the reproduction of discrimination in organizational life (Bjerkestrand et al.; Yang et al.). Importantly, research also highlights how methodological choices shape our understanding of inequality: Sharaf and Shahen show that apparent gender gaps in job satisfaction may disappear once sample selection bias is accounted for. This underscores the need for robust methodological approaches, since without them, inequality may be misinterpreted or overlooked especially in contexts of uneven labor market participation.
